# Sample size and power calculations for open cohort longitudinal cluster randomized trials

**DOI:** 10.1002/sim.8519

**Published:** 2020-03-04

**Authors:** Jessica Kasza, Richard Hooper, Andrew Copas, Andrew B. Forbes

**Affiliations:** ^1^ School of Public Health and Preventive Medicine Monash University Melbourne Victoria Australia; ^2^ Centre for Primary Care and Public Health Queen Mary University of London London UK; ^3^ MRC Clinical Trials Unit University College London London UK

**Keywords:** cluster crossover trial, intra cluster correlation, mixed effects models, stepped wedge

## Abstract

When calculating sample size or power for stepped wedge or other types of longitudinal cluster randomized trials, it is critical that the planned sampling structure be accurately specified. One common assumption is that participants will provide measurements in each trial period, that is, a closed cohort, and another is that each participant provides only one measurement during the course of the trial. However some studies have an “open cohort” sampling structure, where participants may provide measurements in variable numbers of periods. To date, sample size calculations for longitudinal cluster randomized trials have not accommodated open cohorts. Feldman and McKinlay (1994) provided some guidance, stating that the participant‐level autocorrelation could be varied to account for the degree of overlap in different periods of the study, but did not indicate precisely how to do so. We present sample size and power formulas that allow for open cohorts and discuss the impact of the degree of “openness” on sample size and power. We consider designs where the number of participants in each cluster will be maintained throughout the trial, but individual participants may provide differing numbers of measurements. Our results are a unification of closed cohort and repeated cross‐sectional sample results of Hooper et al (2016), and indicate precisely how participant autocorrelation of Feldman and McKinlay should be varied to account for an open cohort sampling structure. We discuss different types of open cohort sampling schemes and how open cohort sampling structure impacts on power in the presence of decaying within‐cluster correlations and autoregressive participant‐level errors.

## 1 INTRODUCTION

**Figure 1:**
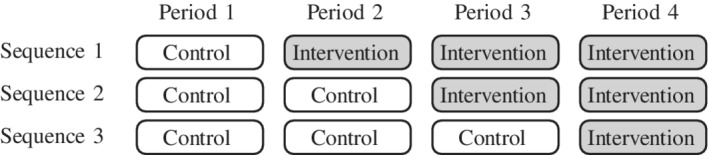
An example stepped wedge schematic, for the stepped wedge design considered in the “Girls on the go!” example in Section 3.1. Multiple clusters may be assigned to each of the treatment sequences

Cluster randomized trials are randomized trials in which clusters of participants, rather than the participants themselves, are randomized to particular treatments.^1^ Longitudinal cluster randomized trials extend standard cluster randomized trials in time: clusters are now randomized to a sequence of treatments, and may switch between intervention and control conditions over the course of the trial.^2^, ^3^ Particular examples of such trials include cluster randomized cross‐over trials,^4^ stepped wedge trials,^5^ or even parallel cluster trial designs, in which measurements are taken at several time points throughout the trial. Figure 1 displays the schematic for an example stepped wedge trial with three treatment sequences. It is well known that the grouping of participants within clusters induces dependence between the measurements taken on different participants within the same cluster. This dependence increases the sample size over that which would be required to detect an effect of the same size in an individually‐randomized trial.^1^ However, longitudinal cluster randomized trials such as the stepped wedge can lead to a reduction in this inflation, by allowing for comparisons within clusters as well as between clusters.^2^, ^6^

When calculating required sample sizes or the power of longitudinal cluster randomized trials, whether individual participants are measured only once or multiple times (once in each of a number of distinct trial periods) must be accounted for. To date, sample size calculations for longitudinal cluster randomized trials have assumed either a closed cohort sampling structure, where all participants contribute measurements in all periods of the trial, or that each participant provides only one measurement. However, as pointed out by Copas et al^7^ in the context of stepped wedge designs, some stepped wedge designs have “open cohort” sampling schemes, where the number of measurements provided by each participant may vary: some participants may provide multiple measurements, and others only one. An example of a stepped wedge design with an open cohort sampling scheme is Tesky et al:^8^ in that trial, nursing homes are the clusters, and residents of the nursing home are recruited to participate in the study. Recruited participants are replaced with new participants if they leave the nursing home, thus maintaining the same sample size in each cluster in each period of the study. Other studies may involve sampling a fixed number of participants from clusters in each period, for example, when the clusters are large communities. When repeated samples are taken from finite populations in this way, it is possible that some participants are sampled and provide measurements in more than one period. A systematic review of stepped wedge trials published between 2010 and 2014 identified 37 stepped wedge trials, with 11 of these having open cohort sampling schemes.^9^ Of these 11 studies, eight provided details on their sample size calculation, all of which appeared to use sample size formulas allowing for one measurement per participant. A more recent review, considering stepped wedge trials published between 2012 and 2017 found 46 studies, of which seven used an open cohort sampling scheme.^10^ Of these seven studies (none of which were included in the Beard et al review), five had prespecified their sample size. It appears that all five of these studies applied sample size formulas appropriate for studies in which each participant provides only one measurement.

Feldman and McKinlay^11^ discussed the possibility of open cohort designs, therein referred to as designs with random overlap, and indicated that the participant autocorrelation (the correlation between mean values of a participant measured at two different time points) could be varied to account for the degree of overlap (the degree of cohort “openness”). In this article, we show precisely how this participant autocorrelation should be varied for open cohort sampling structures, and that this depends on the expected proportion of participants that will be observed in pairs of treatment periods. We provide sample size formulas for open cohort longitudinal cluster randomized trials, with particular emphasis on the stepped wedge design. For the Hussey and Hughes and block‐exchangeable within‐cluster correlation structures, we provide a design effect that unifies the closed cohort and single‐measurement‐per‐participant design effects of Hooper et al.^2^ Our general formulation allows for participants to enter and leave the trial repeatedly; however, in our discussion of different open cohort sampling schemes, we focus on situations where participants do not return after leaving the trial. We also consider the impact of open cohorts on sample size calculations when the correlations of measurements taken from the same or different participants decay the further apart in time measurements are taken. Readers can explore our results in an online app written using R Shiny,^12^ available at https://monash‐biostat.shinyapps.io/OpenCohort/.

## 2 SAMPLE SIZE FORMULAS FOR OPEN COHORT LONGITUDINAL CLUSTER RANDOMIZED TRIALS

### 2.1 Model for open cohort cluster randomized trials

We initially consider a model for a continuous outcome with the block exchangeable within‐cluster correlation structure:^11^ this structure implies that participants measured in the same cluster and the same period of a study have outcomes that are more highly correlated than those of participants measured in the same cluster but in different study periods. While we suppose that the same number of participants is measured in each cluster‐period cell of the trial, we do not necessarily suppose that all of the participants provide measurements in all of the periods. Letting *Y*_*kti*_ be the outcome for participant *i* in period *t* in cluster *k*,

(1) $$\begin{array}{llll} Y_{kti} & =\beta_{t}+\theta X_{kt}+C_{k}+CP_{kt}+\eta_{ki}+\epsilon_{kti},\; & & \\ \eta_{ki} & ∼N(0,\sigma_{\eta }^{2}),\;\epsilon_{kti}∼N(0,\sigma_{\epsilon }^{2}),\;C_{k}∼N(0,\sigma_{C}^{2}),\;CP_{kt}∼N(0,\sigma_{CP}^{2}), \end{array}$$

where participant *i*=1,…,*m*, period *t*=1,…,*T*, cluster *k*=1,…,*K*. Fixed effects for each period are included (the β_*t*_), and previous work has shown that for many longitudinal cluster‐period trials in which all clusters provide measurements in all periods, the variance of the treatment effect estimator (the key ingredient in sample size and power calculations) is invariant to several choices of parameterisation of these time effects.^13^ Participant‐level errors *ϵ*_*kti*_ are assumed to be normally distributed, and the participant‐level random effect η_*ki*_ allows for dependence between multiple measurements on the same participant. Cluster‐level random effects *C*_*k*_ and cluster‐period level random effects *CP*_*kt*_ allow for the correlations between participants measured in the same cluster and the same period to differ from the correlations between participants in the same cluster but different periods. We consider more complex within‐cluster correlation structures and autoregressive participant‐level errors in Section 2.4.

We assume that *m* participants are included in each period in each cluster; however, we do not require that all participants contribute measurements in all periods: there is expected to be some flow of participants into and out of each cluster at each period. Such a situation may be expected in longitudinal cluster randomized trials conducted in schools or residential care facilities, or when cluster members are sampled at each period. In these settings, clusters are expected to maintain a relatively stable cluster size throughout the trial duration, but some participants may leave their cluster and be replaced by new participants during the trial. We do not consider the situation in which participants move from one trial cluster to another: in this situation, there would no longer be independence of outcomes between clusters. We suppose that missing observations from participants who do not provide measurements in all periods are missing at random conditional on the measurements obtained for each individual, or missing completely at random. This implies that we do not consider the implications of informative participant departure, where the very fact that a participant is no longer contributing measurements may provide information about those measurements, or where survivor average causal effects may be of interest.

We consider models with *Y*_*kti*_ that are “within‐period exchangeable”: that is, reordering the *Y*_*kti*_ within periods leads to the same distribution for the vector of *Y*_*kti*_. The theorem in the Supplementary Appendix of Grantham et al^14^ then states that cluster‐period means ${\bar{Y}}_{kt•}=\frac{1}{m}\sum_{i=1}^{m}Y_{kti}$ are a sufficient statistic for θ. Collapsing to cluster‐period means, ${\bar{Y}}_{kt•}=\frac{1}{m}\sum_{i=1}^{m}Y_{kti}$, gives:

(2) $$\begin{array}{llll} {\bar{Y}}_{kt•} & =\beta_{t}+\theta X_{kt}+C_{k}+CP_{kt}+\eta_{k•}+\epsilon_{kt•},\; & & \\ \eta_{k•} & ∼N(0,\sigma_{\eta }^{2}/m),\;\epsilon_{kt•}∼N(0,\sigma_{\epsilon }^{2}/m),\;C_{k}∼N(0,\sigma_{C}^{2}),\;CP_{kt}∼N(0,\sigma_{CP}^{2}). \end{array}$$

Considering the variances and covariances of cluster‐period means shows how this model depends on the open cohort sampling structure:

$$\begin{array}{ll} \mathrm{var}\left({\bar{Y}}_{kt•}\right)=\sigma_{C}^{2}+\sigma_{CP}^{2}+\frac{\sigma_{\eta }^{2}}{m}+\frac{\sigma_{\epsilon }^{2}}{m},\;cov\left({\bar{Y}}_{kt•},{\bar{Y}}_{ks•}\right)=\sigma_{C}^{2}+\sigma_{\eta }^{2}\frac{n_{k}(t,s)}{m^{2}}, & \end{array}$$

where *n*_*k*_(*t*,*s*) is the number of participants in cluster *k* that provide measurements in both periods *t* and *s*, *n*_*k*_(*t*,*s*)=*n*_*k*_(*s*,*t*), *n*_*k*_(*t*,*s*)≤*m* for all period pairs *t*, *s*, and *n*_*k*_(*t*,*t*)=*m*. In order to be valid, each triple of periods *t*, *s*, and *u* must have overlapping numbers of participants that satisfy the following inequality: *n*_*k*_(*t*,*u*)+*n*_*k*_(*u*,*s*)≤*n*_*k*_(*t*,*s*)+*m*. A proof of this requirement is provided in the Appendix. The theory allows for any patterns of participation in the trial, and although the restriction on the *n*_*k*_(*t*,*s*) has no implications for the analysis of open cohort sampling schemes, it does have consequences for the simulation of open cohorts. For some trials, some participants may provide measurements in nonconsecutive sets of periods: this statistical model allows for such participation patterns, but in this article, we consider a more limited set of open cohort sampling schemes, described in Section 2.2.

In some situations, researchers may be aware of how many participants are expected to provide measurements in all pairs of periods of a trial or may wish to reduce response burden by restricting the number of measurements required of each participant, and thus may be able to specify *n*_*k*_(*t*,*s*) for all clusters *k* and period pairs (*t*,*s*). However, when there is uncertainty regarding which participants will be present in each pair of trial periods, researchers may instead have some idea of the rate of participant retention, or equivalently, of participant attrition. The rate of attrition is sometimes referred to as the churn rate, where the churn rate from period *t* to period *s* in cluster *k* is the proportion of participants in period *t* who do not also appear in period *s*:

$$\chi_{k}(t,s)=1-\frac{n_{k}(t,s)}{m}=1-r_{k}(t,s),$$

where *r*_*k*_(*t*,*s*) is the retention rate in cluster *k* between periods *t* and *s*. The covariance between any pair of cluster‐period means depends on the churn rate:

(3) $$\begin{array}{ll} \text{cov}\left({\bar{Y}}_{kt•},{\bar{Y}}_{ks•}|\chi_{k}(t,s)\right)=\sigma_{C}^{2}+\sigma_{\eta }^{2}\frac{1}{m}(1-\chi_{k}(t,s)). & \end{array}$$

Derivations of this and the results in Sections 2.2 and 2.3 are provided in the accompanying Supplementary Material. In some situations, it is reasonable to suppose that χ_*k*_(*t*,*s*)=χ_*k*_ for all period pairs *t*,*s*, and further, that churn rates are constant across clusters, with χ_*k*_=χ, or perhaps *E*[χ_*k*_]=χ. In the next subsection, we will discuss when these assumptions will be appropriate through a discussion of open cohort sampling processes.

### 2.2 Open cohort sampling schemes

**Figure 2:**
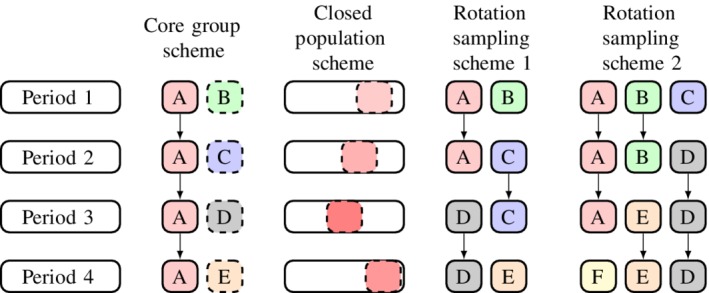
The three variants of open cohort sampling schemes that we will consider, illustrated for a four‐period design. Groups of participants measured in multiple periods are denoted with repeated letters and colours. Rotation sampling scheme 1 has an in‐for‐2 sampling structure, and rotation sampling scheme 2 has an in‐for‐3 sampling structure [Colour figure can be viewed at http://wileyonlinelibrary.com]

There are many different ways in which an open cohort sampling scheme can be realized, and here we discuss three types of schemes that keep *m* constant. Figure 2 displays four specific schemes for a four‐period design. We will discuss each scheme in greater detail, but first summarize each briefly: the “core group” scheme involves a core group of participants in each cluster who provide measurements in each of the periods of the study, complemented by participants who provide measurements in only one period; the “closed population” scheme involves repeated sampling from a closed population of potential study participants; and “rotation sampling” schemes place an upper limit on the number of consecutive periods in which participants will provide measurements and specifies a replacement fraction in each period. The replacement fraction is the proportion of participants who will be be replaced by new participants at the start of each new study period.

In the core group scheme, the churn rate is constant for each pair of periods and can take values χ_*k*_(*t*,*s*)=χ_*k*_∈{0,1/*m*,2/*m*,…,(*m*−1)/*m*,1}. Such a scheme may be appropriate when calculating sample sizes or power for trials taking place in schools or nursing homes, where it is expected that most participants remain in the school or nursing home for the entire duration of the trial, while some may only be present in one trial period. The closed population scheme will be appropriate whenever taking repeated samples from each cluster in each trial period, where some participants may be sampled in multiple periods. Since the sampling is random, at the planning stage of the trial, the expected churn rate is most informative, and will be constant for any pair of periods, *E*[χ_*k*_(*t*,*s*)]=*E*[χ_*k*_]. If the total population is of size *M*, the expected overlapping number of participants between any two periods will be $\frac{m^{2}}{M}$, giving an expected churn rate of $E[\chi_{k}]=1-\frac{m}{M}$.

The rotation sampling scheme has been explored in the context of the design of surveys conducted over multiple time periods, where repeated samples are taken from some population.^15^ We consider rotation designs where each participant provides measurements in a maximum of *p* periods each, with 1/*p* participants in period *t* being replaced by new participants in period *t*+1. This has been referred to as the “in‐for‐*p*” design in the context of surveys.^15^ Rotation sampling scheme 1 in Figure 2 has *p*=2: of the *m* participants who provide measurements in period 1, half will also provide measurements in period 2 (group A), while the other half (group B) will be replaced by group C. Rotation sampling scheme 2 has *p*=3.

For such rotation sampling schemes as this, the churn rate is nonconstant across period pairs. For an “in‐for‐*p*” rotation sampling scheme,

$$\chi_{k}(t,s)=\frac{\left|t-s\right|}{p}\;\text{for}\;|t-s|\leq p\;\text{and}\;1\;\text{for}\;|t-s|>p.$$

Other more complex rotation sampling schemes are possible and have been described in the context of repeated surveys in Steel and McLaren.^15^ For example, participants could be sampled for *p* periods, excluded for *p′* periods, and then return for *p′′* periods.

For core group or closed population schemes, the churn rate does not depend on the length of time between periods, but may differ between clusters. However, if researchers expect that the churn of participants will be similar across clusters, then χ_*k*_=χ can be substituted into Equation (3). Alternatively, researchers may instead expect that the churn rate associated with a given cluster is drawn from some distribution of churn rates, with some clusters having greater churn than others. If all χ_*k*_ are independent and identically distributed with some probability density function *f*_*X*_(χ), this implies that

$$\begin{array}{ll} \text{cov}\left({\bar{Y}}_{kt•},{\bar{Y}}_{ks•}\right)=\sigma_{C}^{2}+\sigma_{\eta }^{2}\frac{1}{m}(1-E\left[\chi_{k}\right]), & \end{array}$$

proof of which is provided in the Supplementary Appendix.

Although the churn rate is, strictly speaking, a discrete random variable, if *m* is large enough, researchers could suppose that the churns follow a Beta distribution, with first parameter α equal to the number of participants expected to be lost from one period to the next averaged over all clusters, and the second parameter β equal to the number of participants retained, again averaged over all clusters. We are assuming that the total number of participants in each cluster‐period is constant, so that α+β=*m*. In that case, χ_*k*_∼*Beta*(α,β), with $E\left[\chi_{k}\right]=\frac{\alpha }{\alpha +\beta }$ and

$$\begin{array}{ll} \text{cov}\left({\bar{Y}}_{kt•},{\bar{Y}}_{ks•}\right)=\sigma_{C}^{2}+\sigma_{\eta }^{2}\frac{1}{m}\frac{\beta }{\alpha +\beta }. & \end{array}$$

The Beta distribution is a convenient choice since it is bounded by 0 and 1, however, all that is required for sample size calculations is the specification of the expected churn rate.

### 2.3 Design effects

Hooper et al^2^ provided design effects for longitudinal cluster randomized trials where participants provide either one measurement only or one measurement in each period of a design (a closed cohort). Here we extend those design effects to incorporate the open cohort sampling scheme when χ_*k*_(*t*,*s*) can be replaced by a constant χ. Following Hooper et al,^2^ we define the following parameters:

(4) $$\begin{array}{ll} \sigma^{2}=\sigma_{C}^{2}+\sigma_{CP}^{2}+\sigma_{\eta }^{2}+\sigma_{\epsilon }^{2},\;\rho =\frac{\sigma_{C}^{2}+\sigma_{CP}^{2}}{\sigma^{2}},\;\pi =\frac{\sigma_{C}^{2}}{\sigma_{C}^{2}+\sigma_{CP}^{2}},\;\tau =\frac{\sigma_{\eta }^{2}}{\sigma_{\eta }^{2}+\sigma_{\epsilon }^{2}}. & \end{array}$$

σ^2^ is the total variance, the parameter ρ is the usual intracluster correlation (the correlation between a pair of participants measured in the same cluster in the same treatment period); π is the cluster autocorrelation (the correlation between two population means from the same cluster measured at different time periods); and τ is the participant/individual autocorrelation (the correlation between two measurements on the same participant in the same period in a given cluster).

If $\theta^{*}$ is the treatment effect that the researcher wishes to detect, with power 1−β and two‐sided significance level α, and the 100*p*th centile of the normal distribution given by *z*_*p*_, then standard results imply that the total number of participants required for an individually randomized trial is

(5) $$\begin{array}{ll} n_{i}=4\frac{\sigma^{2}}{{\left(\theta^{*}\right)}^{2}}{\left(z_{1-\alpha /2}+z_{1-\beta }\right)}^{2}. & \end{array}$$

As has been shown in Hooper et al,^2^ for example, the number of clusters (*K*_*P*_) required for a parallel cluster randomized trial with one measurement taken from each of *m* participants within each cluster is given by

(6) $$\begin{array}{ll} K_{P}=\left[1+(m-1)\rho \right]\frac{n_{i}}{m}, & \end{array}$$

where the quantity 1+(*m*−1)ρ is the design effect that accounts for the clustering of participants.

To account for multiple measurements per cluster, Hooper et al^2^ showed that an additional design effect is required (where the parameter *r* is defined below), given by

(7) $$\begin{array}{ll} DE(r)=\frac{1}{4}\frac{K^{2}(1-r)[1+(T-1)r]}{KX_{••}-\overset{T}{\underset{t=1}{\sum }}{\left(X_{•t}\right)}^{2}+\left[{\left(X_{••}\right)}^{2}+K(T-1)X_{••}-(T-1)\overset{T}{\underset{t=1}{\sum }}{\left(X_{•t}\right)}^{2}-K\overset{K}{\underset{k=1}{\sum }}{\left(X_{k•}\right)}^{2}\right]r}, & \end{array}$$

where *K* is the total number of sequences, and all clusters are assumed to be assigned to their own sequence, *T* is the total number of measurement periods, and

$$\begin{array}{ll} X_{••}=\overset{K}{\underset{k=1}{\sum }}\overset{T}{\underset{t=1}{\sum }}X_{kt},\;X_{•t}=\overset{K}{\underset{k=1}{\sum }}X_{kt},\;X_{k•}=\overset{T}{\underset{t=1}{\sum }}X_{kt}. & \end{array}$$

For the open cohort design, when χ_*k*_(*t*,*s*) can be replaced by a constant χ,

$$r=\frac{\sigma_{C}^{2}+\frac{\sigma_{\eta }^{2}}{m}(1-\chi )}{\sigma_{C}^{2}+\sigma_{CP}^{2}+\frac{\sigma_{\eta }^{2}}{m}+\frac{\sigma_{\epsilon }^{2}}{m}}.$$

This can be written in terms of the correlation parameters as

(8) $$\begin{array}{ll} r=\frac{m\rho \pi +(1-\rho )\tau (1-\chi )}{1+(m-1)\rho }, & \end{array}$$

and can be interpreted as the correlation between two cluster‐period means from the same cluster. This unifies the cross‐sectional and closed cohort design effects in Hooper et al:^2^ when χ=0 the result for closed cohorts is returned, and when χ=1, the result when each participant provides only one measurement is returned.

In Feldman and McKinlay,^11^ a similar unifying model that encompasses cross‐sectional and closed cohort designs was presented. In that, the authors stated that by allowing the participant autocorrelation (which we have here denoted by τ) to vary, their model allowed for “randomly overlapping samples”, and that in the case of overlapping samples, τ will be “small but positive, depending on the degree of the overlap.” Our result shows exactly how the participant autocorrelation should be varied to allow for open cohorts: the participant autocorrelation τ is simply multiplied by the expected retention rate or overlap between periods (ie, proportion of participants expected to be present in both of any pair of periods).

For open cohort longitudinal cluster randomized trials, the number of clusters required is thus given by

(9) $$\begin{array}{ll} K_{L}=DE(r)\times \left[1+(m-1)\rho \right]\frac{n_{i}}{m}, & \end{array}$$

where *n*_*i*_ is the total number of participants required for an individually‐randomized trial, given by Equation (5), *m* is the number of participants measured in each cluster in each period, and ρ is the intracluster correlation for a pair of participants measured in the same cluster period. *DE*(*r*) is given by Equation (7) and depends on the design schematic. The parameter *r*, given in Equation (8), depends on the correlation parameters and the proportion of participants expected to be present in any pair of periods. Dividing *K*_*L*_ by the number of sequences in the design and rounding up to the nearest integer then gives the minimum number of clusters per sequence required to reach the desired level of power. The derivation of *DE*(*r*) is shown in the Supplementary Appendix.

### 2.4 Incorporating decays in between‐period correlations and participant‐level correlations

We consider a model allowing for more general within‐cluster and within‐participant correlation structures. This model includes cluster‐period random effects and correlated participant‐level errors and has the following form:

(10) $$\begin{array}{llll} Y_{kti} & =\beta_{t}+\theta X_{kt}+CP_{kt}+\epsilon_{kti},\; & & \\ \epsilon_{ki} & ={\left(\epsilon_{k1i},\ldots ,\epsilon_{kTi}\right)}^{T}∼N(0,\sigma_{\epsilon D}^{2}D_{\epsilon ,i}),\;CP_{k}={\left(CP_{k1},\ldots ,CP_{kT}\right)}^{T}∼N(0,\sigma_{CP,D}^{2}D_{CP}), \end{array}$$

where *D*_*ϵ*,*i*_ and *D*_*CP*_ are symmetric *T*×*T* matrices with diagonal elements all equal to 1. If participant *i* provides measurements in only *T*_*i*_ periods of the design, then *D*_*ϵ*,*i*_ has dimension *T*_*i*_×*T*_*i*_. We suppose that the elements of *D*_*ϵ*,*i*_ are common across participants: if both participant *i* and *j* provide measurements in periods *t* and *s*, then *D*_*ϵ*,*i*_(*t*,*s*)=*D*_*ϵ*,*j*_(*t*,*s*), and we remove the participant subscript on *D*_*ϵ*_. If *D*_*ϵ*_(*t*,*s*)=τ and *D*_*CP*_(*t*,*s*)=π for *t*≠*s* and some constants τ and π (analogous to the participant and cluster autocorrelations in Equation (4)), then Model (1) is returned. Autoregressive errors at the participant level can be obtained by setting $D_{\epsilon }(t,s)=\tau_{D}^{\left|t-s\right|}$, and the discrete‐time decay model of Kasza et al^16^ is returned if $D_{CP}(t,s)=\pi_{D}^{\left|t-s\right|}$ for constants τ_*D*_ and π_*D*_. Li^17^ presented a similar model for closed‐cohort longitudinal cluster randomized trials with autoregressive participant‐level errors and decaying between‐period correlations.

Collapsing to cluster‐period means gives

$$\begin{array}{llll} {\bar{Y}}_{kt•} & =\beta_{t}+\theta X_{kt}+CP_{kt}+\epsilon_{kt•},\;CP_{k}={\left(CP_{k1},\ldots ,CP_{kT}\right)}^{T}∼N(0,\sigma_{CP,D}^{2}D_{CP}),\; & & \\ \epsilon_{kt•} & =\frac{1}{m}\overset{m}{\underset{i=1}{\sum }}\epsilon_{kti},\;\mathrm{var}(\epsilon_{kt•})=\frac{\sigma_{\epsilon D}^{2}}{m},\;\text{cov}(\epsilon_{kt•},\epsilon_{ks•})=\frac{n_{k}(t,s)}{m^{2}}\sigma_{\epsilon D}^{2}D_{\epsilon }(t,s).\; & & \end{array}$$

As has been shown previously (eg, Kasza et al^18^), the variance of the generalized least‐squares estimator of θ is given by:

$$\begin{array}{ll} \mathrm{var}\left(\hat{\theta }\right)={\left\{\overset{K}{\underset{k=1}{\sum }}X_{k}^{T}\mathrm{var}{\left({\bar{Y}}_{k}\right)}^{-1}X_{k}-\overset{K}{\underset{k=1}{\sum }}X_{k}^{T}\mathrm{var}{\left({\bar{Y}}_{k}\right)}^{-1}{\left[\overset{K}{\underset{k=1}{\sum }}\mathrm{var}{\left({\bar{Y}}_{k}\right)}^{-1}\right]}^{-1}\overset{K}{\underset{k=1}{\sum }}\mathrm{var}{\left({\bar{Y}}_{k}\right)}^{-1}X_{k}\right\}}^{-1}, & \end{array}$$

where $X_{k}^{T}=\left(X_{k1},\ldots ,X_{kT}\right)$ is the vector of treatment assignments for cluster *k*, and $\mathrm{var}({\bar{Y}}_{k})$ is the *T*×*T* variance matrix of the vector ${\bar{Y}}_{k}={\left({\bar{Y}}_{k1•},\ldots ,{\bar{Y}}_{kT•}\right)}^{T}$ with elements

$$\begin{array}{ll} \mathrm{var}({\bar{Y}}_{kt•})=\sigma_{CP,D}^{2}+\frac{\sigma_{\epsilon D}^{2}}{m},\;\text{cov}({\bar{Y}}_{kt•},{\bar{Y}}_{ks•})=\sigma_{CP,D}^{2}D_{CP}(t,s)+\frac{n_{k}(t,s)}{m^{2}}\sigma_{\epsilon D}^{2}D_{\epsilon }(t,s). & \end{array}$$

We provide an online app to allow the calculation of power and sample size for open cohort studies that allows for discrete‐time decays in correlations of cluster and participant random effects, with $D_{CP}(t,s)=\pi_{D}^{\left|t-s\right|}$, and $D_{\epsilon }(t,s)=\tau_{D}^{\left|t-s\right|}$ for constant churn rates. The quantities π_*D*_ and τ_*D*_ are analogous to the parameters π (the cluster autocorrelation) and τ (the participant autocorrelation) in Equation (4). However, π_*D*_ and τ_*D*_ now represent the decay in correlation between cluster or participant random effects for measurements only one period apart in time, rather than the decay in correlation for any pair of measurements from different periods. Users also input the total variance $\sigma_{D}=\sigma_{CP,D}^{2}+\sigma_{\epsilon D}^{2}$ and the intracluster correlation for a pair of measurements in the same cluster in the same period, $\rho_{D}=\frac{\sigma_{CP,D}^{2}}{\sigma_{CP,D}^{2}+\sigma_{\epsilon D}^{2}}$.

### 2.5 Sample size and power for rotation “in‐for‐*p*” open cohort sampling schemes

When the open cohort sampling scheme has an “in‐for‐*p*” structure, $\mathrm{var}({\bar{Y}}_{k})$ is the *T*×*T* variance matrix of the vector ${\bar{Y}}_{k}={\left({\bar{Y}}_{k1•},\ldots ,{\bar{Y}}_{kT•}\right)}^{T}$ with elements

$$\begin{array}{ll} \mathrm{var}({\bar{Y}}_{kt•})=\sigma_{CP,D}^{2}+\frac{\sigma_{\epsilon D}^{2}}{m},\;\text{cov}({\bar{Y}}_{kt•},{\bar{Y}}_{ks•})=\sigma_{CP,D}^{2}D_{CP}(t,s)+\frac{1}{m}\sigma_{\epsilon D}^{2}D_{\epsilon }(t,s)1(|t-s|\leq p)\left(1-\frac{\left|t-s\right|}{p}\right), & \end{array}$$

where 1(|t−s|≤p) is the indicator function for the event |*t*−*s*|≤*p*. The online app allows calculation of sample size and power when in‐for‐*p* sampling schemes are of interest. Users can select the sampling scheme, and when selecting in‐for‐*p*, the power or sample size for values of *p*=1,…,*T*, where *T* is the number of periods in the user‐input design are graphed.

## 3 EXAMPLES OF SAMPLE SIZE CALCULATIONS WITH OPEN COHORTS

### 3.1 Girls on the Go! example

We consider a specific example inspired by the closed‐cohort example described in Hooper et al:^2^ a stepped wedge trial conducted in Australian primary schools to evaluate the “Girls on the go!” program aimed at increasing the self‐esteem of young women.^19^ The primary outcome was the Rosenberg Self‐esteem scale, a continuous measure. Following Hooper et al,^2^ we assume an intracluster correlation of ρ=0.33, a cluster autocorrelation of π=0.9, an individual autocorrelation of τ=0.7, a total variance of 25, and a mean difference of interest of 2 units. The standard three‐sequence stepped wedge design was implemented, as shown in Figure 1, with two schools assigned to each of the three sequences in the original trial, with 10 students enrolled in each school. We suppose here that four schools were assigned to each of the three sequences: Hooper et al^2^ showed that such a study would have a power of 89.3%. In reality, this study had a closed cohort sampling scheme, but we investigate the impact of a core group open cohort sampling scheme, assuming that the core group made up 0%, 10%, 20%, …, 100% of the sample in each cluster, and of in‐for‐*p* sampling schemes, with *p*=1,2,3,4.

**Figure 3:**
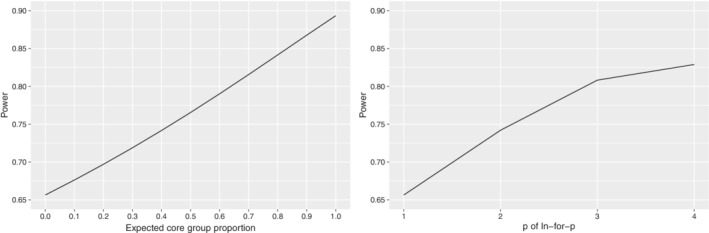
Power for the “Girls on the go!” program, for varying expected core group proportion (left panel); and varying *p* of in‐for‐*p* sampling schemes (right panel). When expected core group proportion is 0, each participant provides only one measurement during the trial; when expected core group proportion is 1, each participant provides one measurement in each trial period. When *p*=1, each participant provides only one measurement during the trial

Figure 3 displays the power for changing core group proportions (left) and changing *p* for in‐for‐*p* sampling schemes (right). As the expected core group proportion or the maximum number of periods in which participants provide measurements in in‐for‐*p* schemes increases, so too does the power of the study. This is to be expected since the estimator for the treatment effect that we consider (the generalized least squares estimator) combines both within‐cluster and between‐cluster comparisons.^6^ As the average number of measurements per participant increases (with increasing core group proportion or increasing *p*), within‐cluster comparisons contribute increasing amounts of information about the treatment effect. When the expected core group proportion is 0 or *p*=1, each participant provides only one measurement over the course of the trial, the two sampling schemes coincide and the power of the study is minimized for this example. When the core group proportion is 1, the core group sampling scheme becomes a closed cohort; however, there is no value of *p* for which the in‐for‐*p* scheme coincides with a closed cohort. When *p*=4, only one quarter of participants recruited in the first period will provide measurements in each period of the trial. In fact, when there is nonzero correlation between measurements on the same participant, the in‐for‐*p* sampling scheme will never be as powerful as a closed cohort: there will always be a proportion of participants that provide measurements in the first period of a study only under the in‐for‐*p* sampling scheme.

### 3.2 Incorporating decaying correlations

We extend the “Girls on the go!” example to include decaying correlations and autoregressive participant‐level errors and consider the impact of participant retention rate on power when there is no decay (the scenario considered in Section 3.1), a decay in the participant‐level correlation only, when there is a decay in the cluster‐level autocorrelation only, and when there is decay in both the participant‐ and cluster‐level correlations. As above, we consider a three‐sequence stepped wedge design with four clusters assigned to each of the three sequences, and 10 participants in each cluster in each period, and a total variance of 25 units with a mean difference of interest of 2 units.

For all four scenarios, the assumed intracluster correlation is given by ρ_*D*_=0.33; however, the cluster autocorrelation and participant autocorrelation selected depend on whether there is supposed to be decay in those correlations. The values τ=0.7 and π=0.9 in Section 3.1 were specified under the assumption that these autocorrelations would specify the decay for any pair of periods, no matter how far apart in time these may be. However, as has been shown in Kasza and Forbes,^20^ if there is a decay in correlations over time, and a model that does not allow for such decay is specified, the estimate of the cluster autocorrelation will account for the decay that was present in the dataset. Hence, when calculating sample sizes where the correlations between cluster‐level or subject‐level random effects decay over time, values of τ and π that were estimated under a model without decay should not be directly substituted: the autocorrelation parameters in the models without decay are not equivalent to the autocorrelation parameters in models with decay. Extending the formulas in Kasza and Forbes,^20^ we derive adjusted values of the cluster and participant autocorrelations, that is, the values of τ_*D*_ and π_*D*_ compatible with the estimates of τ and π obtained from the misspecified model that fails to account for the decay in autocorrelations over time. This amounts to solving the equations

$$\overset{T}{\underset{t=1}{\sum }}\overset{T}{\underset{s=1}{\sum }}\tau_{D}^{\left|t-s\right|}=\tau T(T-1)+T\;\text{and}\;\overset{T}{\underset{t=1}{\sum }}\overset{T}{\underset{s=1}{\sum }}\pi_{D}^{\left|t-s\right|}=\pi T(T-1)+T$$

for τ_*D*_ and π_*D*_. Doing this for *T*=4, τ=0.7 and π=0.9 gives τ_*D*_=0.80 and π_*D*_=0.94. Only when decaying cluster‐level autocorrelations are incorporated is the value π_*D*_ is assumed, and only when decaying participant‐level autocorrelations are incorporated is the value τ_*D*_; otherwise, π and τ are included in calculations. In the online app, we have assumed that users will input the values of autocorrelation parameters that were estimated under the same model they assume for future trial data. However, if users only have values of τ and π that were estimated from a model without decay over time but wish to consider the impact of such a decay on their study power, they can first apply the formulas above to obtain adjusted values of τ and π (ie, τ_*D*_ and π_*D*_), and input these adjusted values into the app.

**Figure 4:**
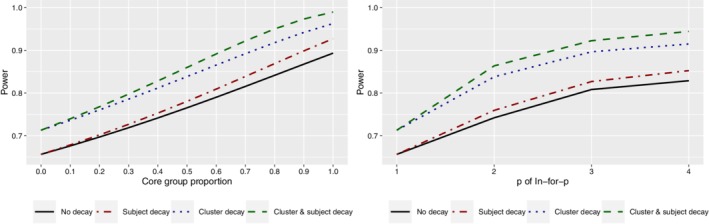
Differences between the theoretical power for the “Girls on the go!” program without any decay in between‐period correlations and participant errors and for models assuming decaying between‐period correlations and/or autoregressive participant errors for differing core group proportions (left) and *p* of in‐for‐*p* sampling schemes (right) [Colour figure can be viewed at http://wileyonlinelibrary.com]

Figure 4 displays the power for each of the four considered correlation structures (no decay; decay in participant autocorrelations only; decay in cluster autocorrelations only; decay in both participant and cluster autocorrelations) for core group proportions from 0 to 1 (left) and for differing rotation sampling schemes (*p*=1,2,3,4 for in‐for‐*p* sampling schemes). For all correlation structures, the power increases as the core group proportion increases or when the maximum number of measurements provided by each participant increases (increasing *p*). For the core group scheme, the steepest increases occur when there is decay in the participant autocorrelation. When a decay in participant autocorrelation is included, the correlation between measurements in successive periods is greater than when there is no decay (τ_*D*_=0.8 versus τ=0.7). The greater the autocorrelation between successive measurements on the same participant, the more information there is in the comparison of outcomes from that participant measured under control and intervention conditions. When the core group proportion is higher, the more participants there are that provide measurements under both control and intervention conditions in successive periods, and thus the power to detect a given effect size increases.

Similarly, power is greater when cluster autocorrelations decay over time than when there is no decay, for all schemes: this is the case because π_*D*_ is greater than π, so when a decaying cluster autocorrelation is accounted for, measurements in the same cluster in adjacent periods are more highly correlated than when there is no decay (π_*D*_=0.94 versus π=0.9).

## 4 DISCUSSION

In this article, we have presented formulas for sample size and power calculations for open cohort longitudinal cluster randomized trials, where participants may provide varying numbers of measurements. Design effects were provided for the model with a block‐exchangeable within‐cluster correlation structure, and a formula for the variance of the treatment effect estimator was provided for when the within‐cluster correlation structure is more complex. The design effect unifies the closed cohort and single‐measurement design effects provided in Hooper et al.^2^ We have also provided an online app to allow readers to investigate the impact of varying degrees of cohort openness on the power of their planned studies.

When the churn rate is constant across clusters and period pairs (eg, for core group and closed population sampling schemes), for designs in which some or all clusters switch between treatments, the conservative assumption is that each participant provides one measurement only: this will always lead to larger sample sizes. Hence, researchers may be tempted to conservatively assume a retention rate of 0 (ie, completely nonoverlapping samples at each study period). However, when planning studies, researchers should carefully consider the ethical implications of exposing participants to involvement in a clinical trial and use a value of the retention rate that accurately reflects what is expected to happen during the trial.

Assuming a common expected retention rate across clusters that does not depend on the time between periods leads to closed‐form sample size formulas. In many situations, such as core group and closed population sampling schemes, we would expect that such an assumption would be adequate, but further work is required to assess the impact of varying retention rates. For example, rotation sampling schemes imply that the churn between two periods, χ_*k*_(*t*,*s*) depends on the amount of time between periods, |*t*−*s*|. Other sampling schemes may also lead to an increase in churn over time. We have only considered three different types of sampling schemes possible in open cohort designs: the core group, closed population, and rotation sampling schemes (specifically, in‐for‐*p* schemes). Other sampling schemes are indeed possible and have been explored at length in the repeated survey literature. It seems that further research into the applicability of alternative open cohort sampling schemes in the context of longitudinal cluster randomized trials is necessary. When researchers wish to minimize the burden on participants, rotation sampling schemes may be a good choice, but further work on these and related schemes is required to determine the impact of changing the number of measurements on subjects for various types of longitudinal cluster randomized trials: increasing the maximum number of periods for which subjects are observed (ie, increasing *p* in “in‐for‐*p*” designs) is not guaranteed to increase the power of the study. Furthermore, some researchers may wish to reduce response burden in participants by sampling subjects in every second period. Further research could also incorporate the differential costs of a repeated measurement on a participant versus that of a measurement of a new participant, particularly in a survey setting where subject‐matter explanation is required as part of the data acquisition process.

Researchers reporting open‐cohort longitudinal cluster randomized trials should be encouraged to report the number of participants overlapping for each pair of periods in each cluster, or at least some estimate of the retention rate. If different clusters have different expected rates of retention, upper and lower bounds on required sample sizes can be obtained by assuming the lowest and the highest expected retention rate across all clusters. We also assumed that the number of participants was the same in each cluster‐period.

The within‐cluster correlation structures we have considered depend on treatment periods, rather than on the specific trial entry time of each participant: time is treated as a discrete phenomenon, taking values 1,2,…,*T*. Recent papers have discussed time as a continuous phenomenon in longitudinal cluster randomized trials, where participants have outcomes that are measured in continuous time, rather than at a set of discrete time points common to all participants. Grantham et al^14^ discussed within‐cluster correlation structures in the context of continuous time, and Hooper and Copas^21^ discussed the need to clarify sampling schemes and the terminology used to refer to specific sampling schemes. If participants can have their observations recorded at any time, rather than at a set of discrete times common to all participants, then the correlation structures we have assumed for cluster‐level random effects are not likely to be satisfactory: these correlation structures imply that all participants within a period are exchangeable, and that any pair of participants measured in a period are more highly correlated than any pair of participants measured in distinct periods. However, it may be more plausible to assume that participants measured at the start and end of a period have outcomes that are less correlated than participants who are measured at the end of one period and the start of the next. Correlation structures such as that described in Grantham et al^14^ imply that outcomes from the same cluster and in the same period are no longer exchangeable. When there is no longer exchangeability within periods, precisely which participants provide measurements in each pair of periods, rather than just the number overlapping in each pair of periods, becomes important for sample size calculations.

In this article, we have provided design effects and sample size formulas for open cohort sampling structures, unifying previous work that provided separate results for closed cohort sampling structures and single‐measurement structures. We have considered three different types of open cohort sampling schemes, but there are likely many more that trialists may find useful. Future work should consider alternative open cohort sampling schemes and the questions of participants moving between clusters. Furthermore, the impact of treatment effect heterogeneity across clusters, and other correlation structures that depend on treatment periods, could be considered in the context of open cohorts.

## CONFLICT OF INTEREST

The authors declare no potential conflict of interests.

## DATA ACCESSIBILITY

Data sharing is not applicable to this article as no new data were created or analyzed in this study. The code for the R Shiny app is available online at https://github.com/jkasza/OpenCohort.

## Supporting information

Data S1: Supplementary material

## APPENDIX A

### A.1

We here prove that if *n*_*k*_(*t*,*s*) is the number of participants providing measurements in both period *t* and period *s*, then *n*_*k*_(*t*,*u*)+*n*_*k*_(*u*,*s*)≤*n*_*k*_(*t*,*s*)+*m*, where *m* is the number of participants observed in each cluster in each period. We consider a particular cluster and omit the cluster subscripts *k*. Suppose that in this cluster, a total of *M* participants provide measurements, and the vector of length *M*, *x*_*t*_=(*x*_*t*1_,…,*x*_*tM*_)^*T*^ indicates whether each participant provides a measurement in period *t*: *x*_*ti*_=1 if participant *i* provides a measurement in period *t*, and *x*_*ti*_=0 if not.

Define $l(t,s)=\sum_{i=1}^{M}|x_{ti}-x_{si}|$: this counts the number of participants that provide measurements in period *t* only or period *s* only and can be thought of as the distance between periods *t* and *s* in terms of participants. *l*(*t*,*s*) is commonly referred to as the taxi‐cab metric, and satisfies the triangle inequality:

l(t,s)≤l(t,u)+l(u,s).

An equivalent definition of *l*(*t*,*s*) is

$$l(t,s)=\overset{M}{\underset{i=1}{\sum }}{\left(x_{ti}-x_{si}\right)}^{2}=\overset{M}{\underset{i=1}{\sum }}\left(x_{ti}^{2}+x_{si}^{2}-2x_{ti}x_{si}\right)=2m-2\overset{M}{\underset{i=1}{\sum }}x_{ti}x_{si}=2m-2n(t,s).$$

Applying the triangle inequality to this definition gives the result.

In fact, all sets of *n*_*k*_(*t*,*s*) that satisfy the given restriction are valid open cohort sampling schemes: if *n*_*k*_(*t*,*u*)+*n*_*k*_(*u*,*s*)≤*n*_*k*_(*t*,*s*)+*m*, then vectors *x*_*t*_ of maximum length *m*×*T*, where *T* is the number of periods and *m* the number of participants in each period can be defined that describe the open cohort sampling scheme.
