# Supplementary material for: Sample size and power calculations for open cohort longitudinal cluster randomized trials
Source: Stat Med. 2020 Mar 4;39(13):1871–83. doi: 10.1002/sim.8519 (PMC7217159; doi:10.1002/sim.8519)
Supplement: Supplementary file 1 — Data S1: Supplementary material [file SIM-39-1871-s001.pdf]

Supplementary Material to accompany: Sample size and power calculations for open cohort longitudinal cluster randomised trials

Jessica Kasza, Richard Hooper, Andrew Copas, Andrew B Forbes

January 16, 2020

1 Derivation of formulas

Y_{kti} represents the outcome for participant i in period t in cluster k,

$$\begin{aligned} &Y_{kti} = \beta_t + \theta X_{kt} + C_k + CP_{kt} + \eta_{ki} + \epsilon_{kti}, \\ &\eta_{ki} \sim N(0, \sigma^2_\eta), \quad \epsilon_{kti} \sim N(0, \sigma^2_\epsilon), \quad C_k \sim N(0, \sigma^2_C), \quad CP_{kt} \sim N(0, \sigma^2_{CP}) \end{aligned} \tag{1}$$

where participant $i = 1, \ldots, m$, period $t = 1, \ldots, T$, cluster $k = 1, \ldots, K$. Fixed effects for each period are included (the β_t). Participant-level errors ϵ_{kti} are assumed to be normally distributed, and the participant-level random effect η_{ki} allows for dependence between multiple measurements on the same participant. Cluster-level random effects C_k and cluster-period level random effects CP_{kt} allow for the correlations between participants measured in the same cluster and the same period to differ from the correlations between participants in the same cluster but different periods. Collapsing to cluster-period means, $\bar{Y}_{kt\bullet} = \frac{1}{m}\sum_{i=1}^m Y_{kti}$, gives:

$$\begin{aligned} &\bar{Y}_{kt\bullet} = \beta_t + \theta X_{kt} + C_k + CP_{kt} + \eta_{k\bullet} + \epsilon_{kt\bullet}, \\ &\eta_{k\bullet} \sim N(0, \sigma^2_\eta/m), \quad \epsilon_{kt\bullet} \sim N(0, \sigma^2_\epsilon/m), \quad C_k \sim N(0, \sigma^2_C), \quad CP_{kt} \sim N(0, \sigma^2_{CP}). \end{aligned} \tag{2}$$

Considering the variances and covariances of cluster-period means shows how this model depends on the open cohort sampling structure:

$$var\left(\bar{Y}_{kt\bullet}\right) = \sigma^2_C + \sigma^2_{CP} + \frac{\sigma^2_\eta}{m} + \frac{\sigma^2_\epsilon}{m}, \quad cov\left(\bar{Y}_{kt\bullet}, \bar{Y}_{ks\bullet}\right) = \sigma^2_C + \sigma^2_\eta \frac{n_k(t,s)}{m^2}$$

where $n_k(t,s)$ is the number of participants in cluster k that provide measurements in both periods t and s, $n_k(t,s) = n_k(s,t)$, $n_k(t,s) \le m$ for all period pairs t, s, and $n_k(t,t) = m$.

We step through the derivation of the formula for $cov\left(\bar{Y}_{kt\bullet}, \bar{Y}_{ks\bullet}\right)$, leading to Equation (3) of the main paper (dropping the fixed effect terms immediately):

$$cov\left(\bar{Y}_{kt\bullet}, \bar{Y}_{ks\bullet}\right) = \frac{1}{m^2} cov\left(\sum_{i=1}^{m} C_k + CP_{kt} + \eta_{ki} + \epsilon_{kti}, \sum_{j=1}^{m} C_k + CP_{ks} + \eta_{kj} + \epsilon_{ksj}\right)$$
$$= \frac{1}{m^2}\left\{m^2 var(C_k) + m^2 cov(CP_{kt}, CP_{ks}) + cov\left(\sum_{i=1}^{m}\eta_{ki}, \sum_{j=1}^{m}\eta_{kj}\right) + cov\left(\sum_{i=1}^{m}\epsilon_{kti}, \sum_{j=1}^{m}\epsilon_{ksj}\right)\right\}.$$

Since $cov(CP_{kt}, CP_{ks}) = 0$ and $cov\left(\epsilon_{kti}, \epsilon_{ksj}\right) = 0$ for $i = j$ or $i \ne j$ (by independence),

$$cov\left(\bar{Y}_{kt\bullet}, \bar{Y}_{ks\bullet}\right) = var(C_k) + \frac{1}{m^2} cov\left(\sum_{i=1}^{m}\eta_{ki}, \sum_{j=1}^{m}\eta_{kj}\right) = \sigma_C^2 + \frac{1}{m^2} cov\left(\sum_{i=1}^{m}\eta_{ki}, \sum_{j=1}^{m}\eta_{kj}\right).$$

Note that the i and j indices on the η_{kti} and η_{ktj} do not necessarily refer to distinct participants. If a participant provides a measurement in both periods t and s, then there will be an i and j such that $cov(\eta_{ki}, \eta_{kj}) = var(\eta_{ki})$. Since there are $n_k(t,s)$ such participants, we get $cov\left(\bar{Y}_{kt\bullet}, \bar{Y}_{ks\bullet}\right) = \sigma_C^2 + \sigma_\eta^2 \frac{n_k(t,s)}{m^2}$.

The covariance between $\bar{Y}_{kt\bullet}$ and $\bar{Y}_{ks\bullet}$ implicitly depends on $n_k(t,s)$ and thus on the churn rate $\chi_k(t,s) = 1 - \frac{n_k(t,s)}{m} = 1 - r_k(t,s)$. If $\chi_k(t,s)$ is a random variable, this dependence should be made explicit, leading to:

$$cov\left(\bar{Y}_{kt\bullet}, \bar{Y}_{ks\bullet} | \chi_k(t,s)\right) = \sigma_C^2 + \sigma_\eta^2 \frac{1}{m}(1 - \chi_k(t,s)),$$

which is Equation (3) in the main paper.

We then obtain the result in Section 2.2 of the main paper. If we can assume that $E[\chi_k(t,s)] = \chi$, then applying the Law of Total Covariance gives

$$cov\left(\bar{Y}_{kt\bullet}, \bar{Y}_{ks\bullet}\right) = \sigma_C^2 + \sigma_\eta^2 \frac{1}{m}(1 - \chi).$$

Finally, we demonstrate how the results in Section 2.3 of the main paper can be obtained, by obtaining an expression for the variance of the treatment effect estimator. If $\bar{Y} = \left(\bar{Y}_{k1\bullet}, \ldots, \bar{Y}_{kT\bullet}\right)^T$, then

$$var\left(\bar{Y}\right) = V = \left(\sigma_{CP}^2 + \sigma_\epsilon^2 \frac{1}{m} + \sigma_\eta^2 \frac{\chi}{m}\right) I + \left(\sigma_C^2 + \sigma_\eta^2 \frac{1}{m}(1 - \chi)\right) J$$

where I is the $T \times T$ identity matrix and J is the $T \times T$ matrix of ones. An expression for $var(\hat{\theta})$, where $\hat{\theta}$ is the generalised least squares estimator used in Hussey and Hughes [2007], can then be obtained. Working through the following expression (from Kasza et al. [2019]):

$$var(\hat{\theta}) = \left(\sum_{k=1}^{K} X_k^T V^{-1} X_k - \frac{1}{K} \left(\sum_{k=1}^{K} X_{k1}, \dots, \sum_{k=1}^{K} X_{kT} \right) V^{-1} \begin{pmatrix} \sum_{k=1}^{K} X_{k1} \\ \vdots \\ \sum_{k=1}^{K} X_{kT} \end{pmatrix} \right)^{-1}$$

where $X_k = (X_{k1}, \dots, X_{kT})^T$ is the vector of treatment assignments for cluster k. First note that $\sigma_{CP}^2 + \sigma_\epsilon^2 \frac{1}{m} + \sigma_\eta^2 \frac{\chi}{m} = \frac{\sigma^2}{m}(1 + (m-1)\rho)(1-r)$ and $\sigma_C^2 + \sigma_\eta^2 \frac{1}{m}(1-\chi) = \frac{\sigma^2}{m} r(1 + (m-1)\rho)$. Standard matrix algebra can be used to show that

$$V^{-1} = \frac{1}{\frac{\sigma^2}{m}(1 + (m-1)\rho)(1-r)} \left(I + \frac{r}{(1 + r(T-1))} \right).$$

Working through the (somewhat tedious but relatively straightforward) algebra (and noting that when X_{kt} is coded as 0/1, $X_{kt}^2 = X_{kt}$) then gives

$$var(\hat{\theta}) = \frac{\sigma^2}{m} \frac{K(1 + (m-1)\rho)(1-r)(1 + r(T-1))}{K X_{\bullet\bullet} - \sum_{t=1}^{T} (X_{\bullet t})^2 + \left[(X_{\bullet\bullet})^2 + K(T-1) X_{\bullet\bullet} - (T-1) \sum_{t=1}^{T} (X_{\bullet t})^2 - K \sum_{k=1}^{K} (X_{k\bullet})^2 \right] r}.$$

Comparing this expression to that obtained for an individually randomised trial with n participants in total ($\frac{4\sigma^2}{n}$) then gives the result in Equation (9) of the main paper.

References

M. A. Hussey and J. P. Hughes. Design and analysis of stepped wedge cluster randomized trials. Contemporary Clinical Trials, 28:182–191, 2007.

J. Kasza, K. Hemming, R. Hooper, J. N. S. Matthews, and A. B. Forbes. Impact of non-uniform correlation structure on sample size and power in multiple-period cluster randomised trials. Statistical Methods in Medical Research, 28(3):703–716, 2019.
